# Supplementary figures and images for: Heligmosomoides bakeri and Toxoplasma gondii co-infection leads to increased mortality associated with changes in immune resistance in the lymphoid compartment and disease pathology
Source: PLoS One. 2024 Jul 1;19(7):e0292408. doi: 10.1371/journal.pone.0292408 (PMC11216590; doi:10.1371/journal.pone.0292408)

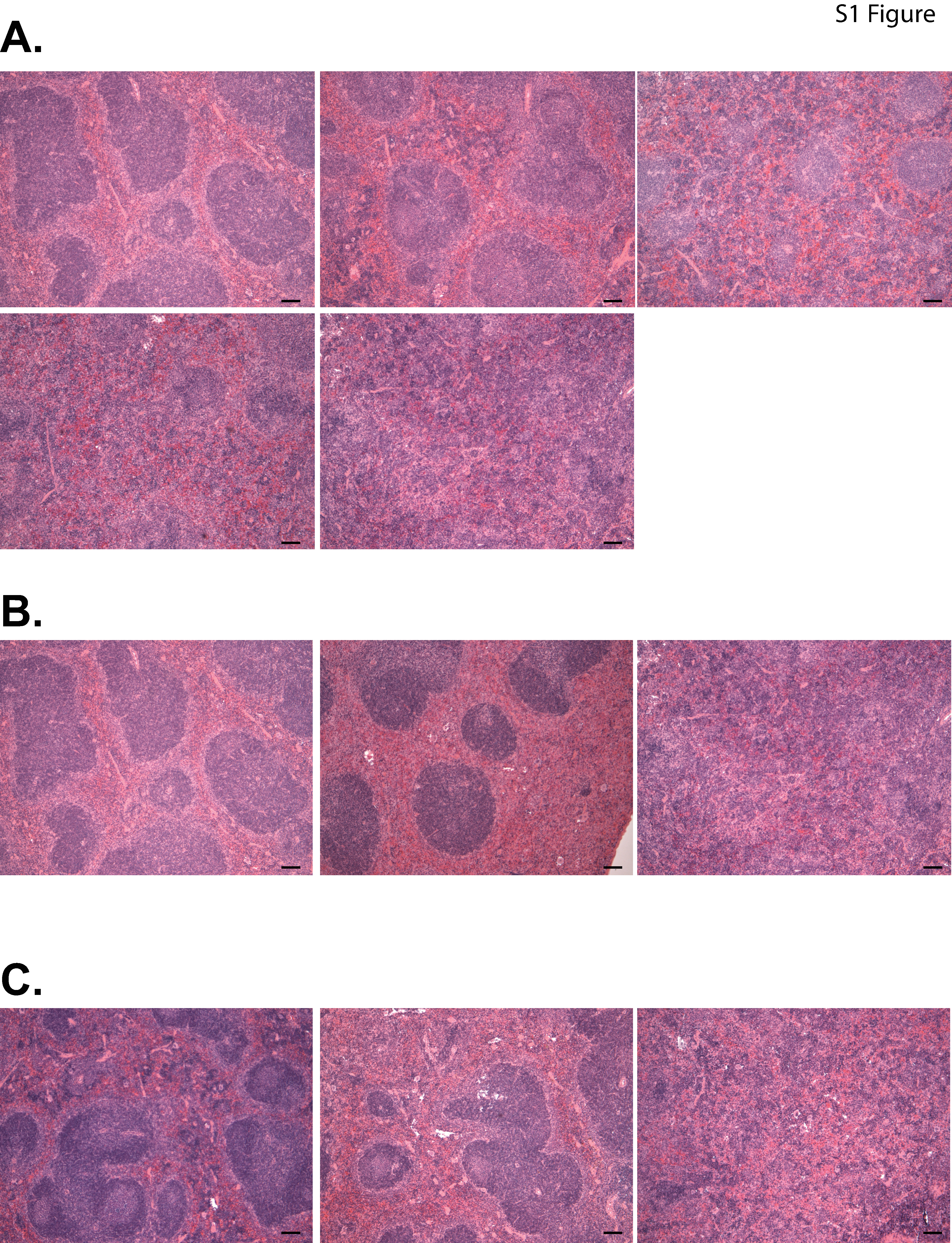

Supplement: S1 Fig — (A). Follicle damage: All follicles are distinct and defined in shape (score of 1, top left). A majority of follicles are distinct and defined in shape (score of 2, top middle). Approximately equal numbers of intact and damaged follicles. (score of 3, top right). The majority of follicles are damaged, with some defined follicles observed (score of 4, bottom left). All follicles are damaged (score of 5, bottom right). Scale 10um. (B) Marginal zone thickness: More than 50% of the follicles have a thick marginal zone (score of 1, left). More than 50% of the follicles have a thin marginal zone (score of 2, middle) More than 50% of the follicles do not have an observable marginal zone (score of 3, right). (C) Size of the light zone of the germinal centre: More than 50% of the follicles have a small germinal center (score of 1, left). More than 50% of the follicles have a large germinal center (score of 2, middle). More than 50% of the follicles have no observable germinal center (score of 3, right). (TIF) [file pone.0292408.s001.tif]

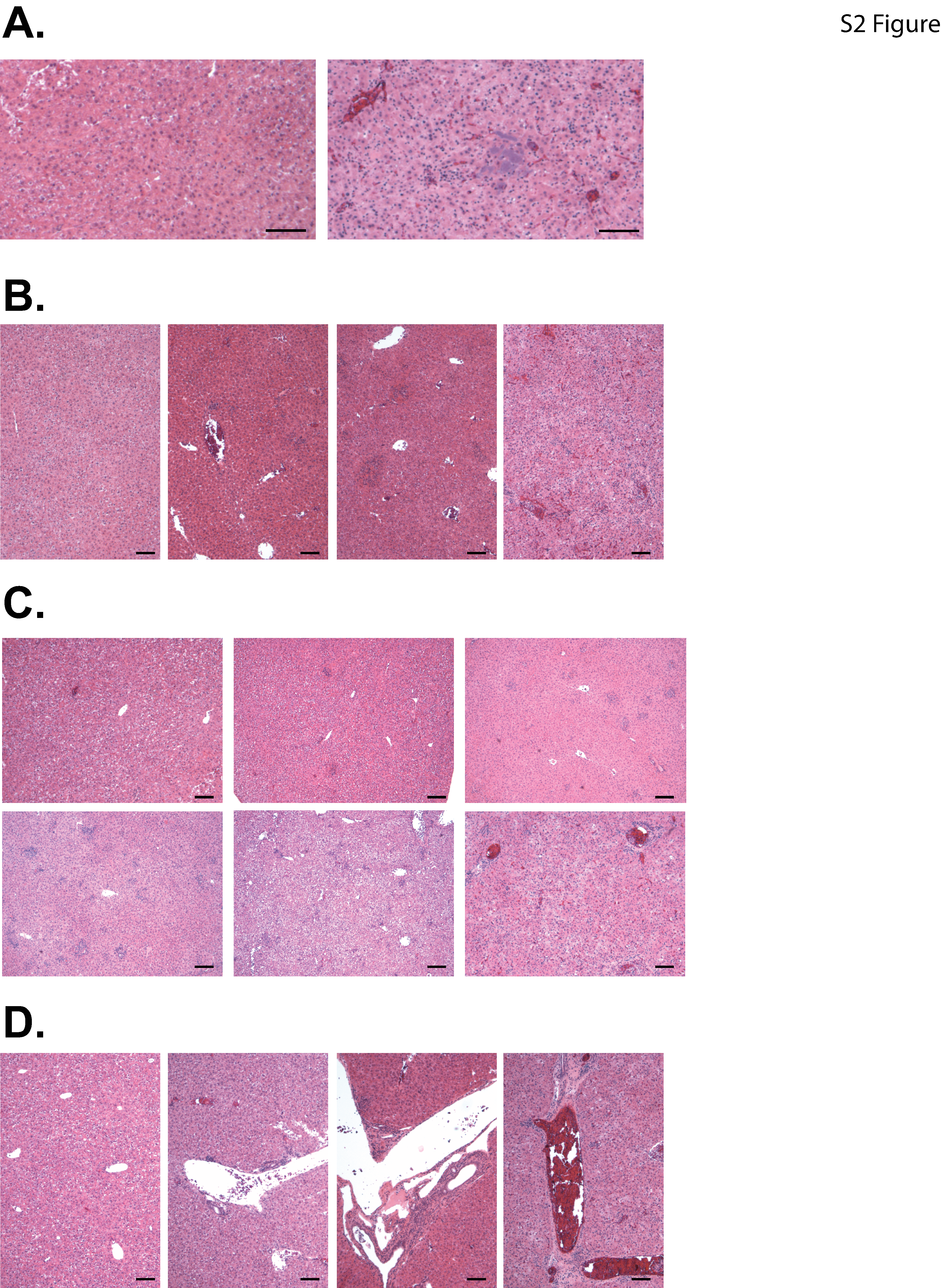

Supplement: S2 Fig — (A). Necrosis: absence (score of 1, left) and presence (score of 2, right) of necrosis. Scale 10um. (B) Infiltrate Size: All infiltrates observed are organized into small groups (score of 1, left), 75% of infiltrates observed are organized into small groups and 25% into large groups (score of 2, middle left), 50/50 split between small and large infiltrate groups (score of 3, middle right), 100% of infiltrates are organized into large groups (score of 4, right). (C). Proportion of infiltrates: very few infiltrate groups are observed (score of 1, top left), some groups of infiltrates are observed (score of 2, top middle), a medium number of infiltrates is observed (score of 3, top right), many infiltrate groups are observed and occupy the majority of the tissue (score of 4, bottom left), infiltrate groups are distinguishable, and occupy most of the tissue (score of 5, bottom middle), and Infiltrate groups are indistinguishable and occupy the entire tissue (score of 6, bottom right). (D) Perivascular infiltrates: Very few infiltrating leukocytes can be observed in the vessels (score of 1, left). Infiltrating leukocytes are observed in small numbers leaving most of the vessels (score of 2, left middle). Most of the vessels are surrounded by infiltrating leukocytes (score of 3, right middle). All of the vessels are filled with infiltrating leukocytes (score of 4, right). (TIF) [file pone.0292408.s002.tif]

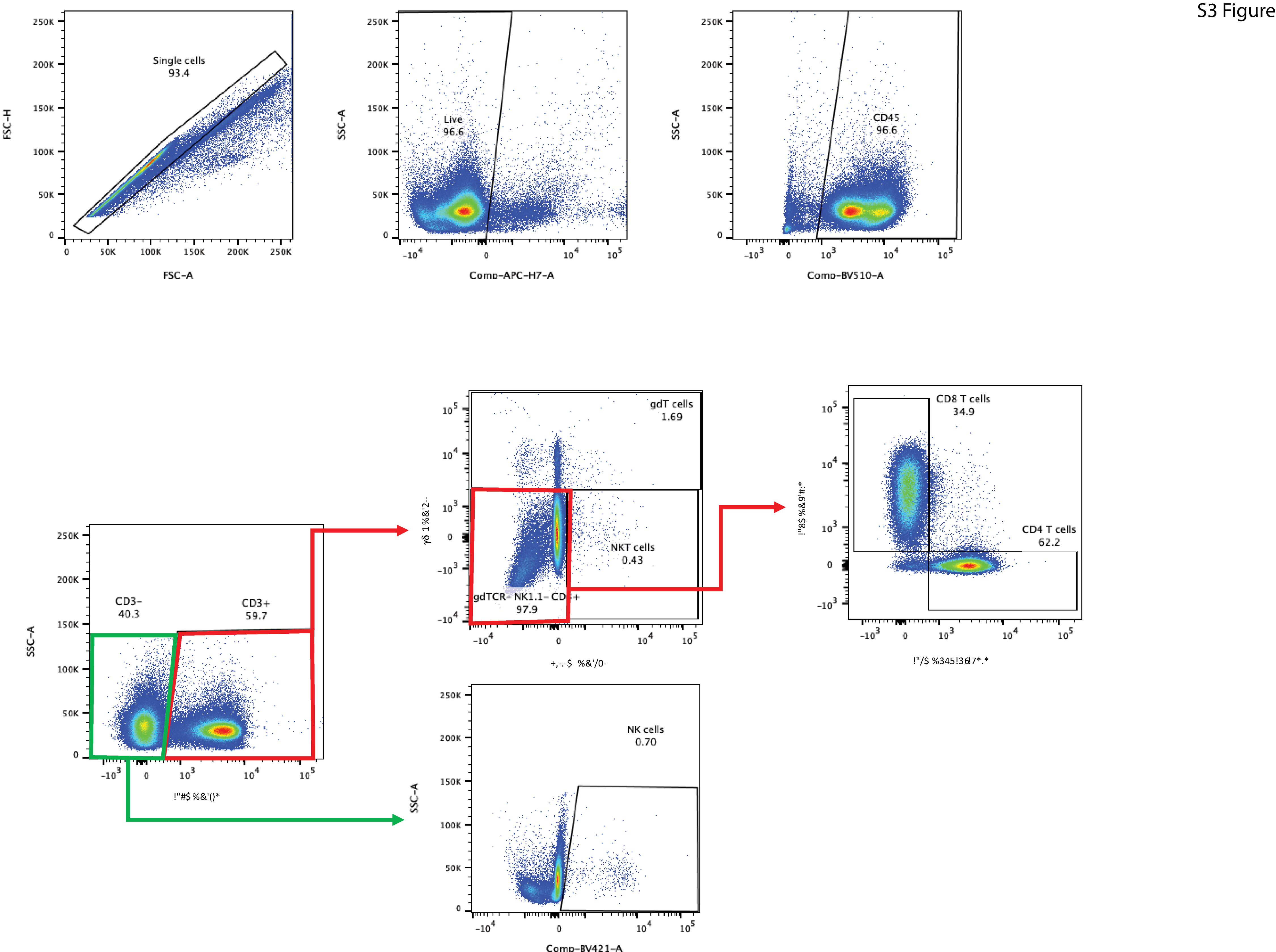

Supplement: S3 Fig — Doublets were removed using the FSC-A and FSC-H parameters. Live cells were selected, followed by CD45+ cells. The CD3- subset was used to identify NK cells using NK1.1. The CD3+ subset was divided into ©δ cells (©δ+), NKT cells (©δ-αβ+NK1.1+) and the αβ cells were subdivided into CD4+ and CD8+ cells. (TIF) [file pone.0292408.s003.tif]

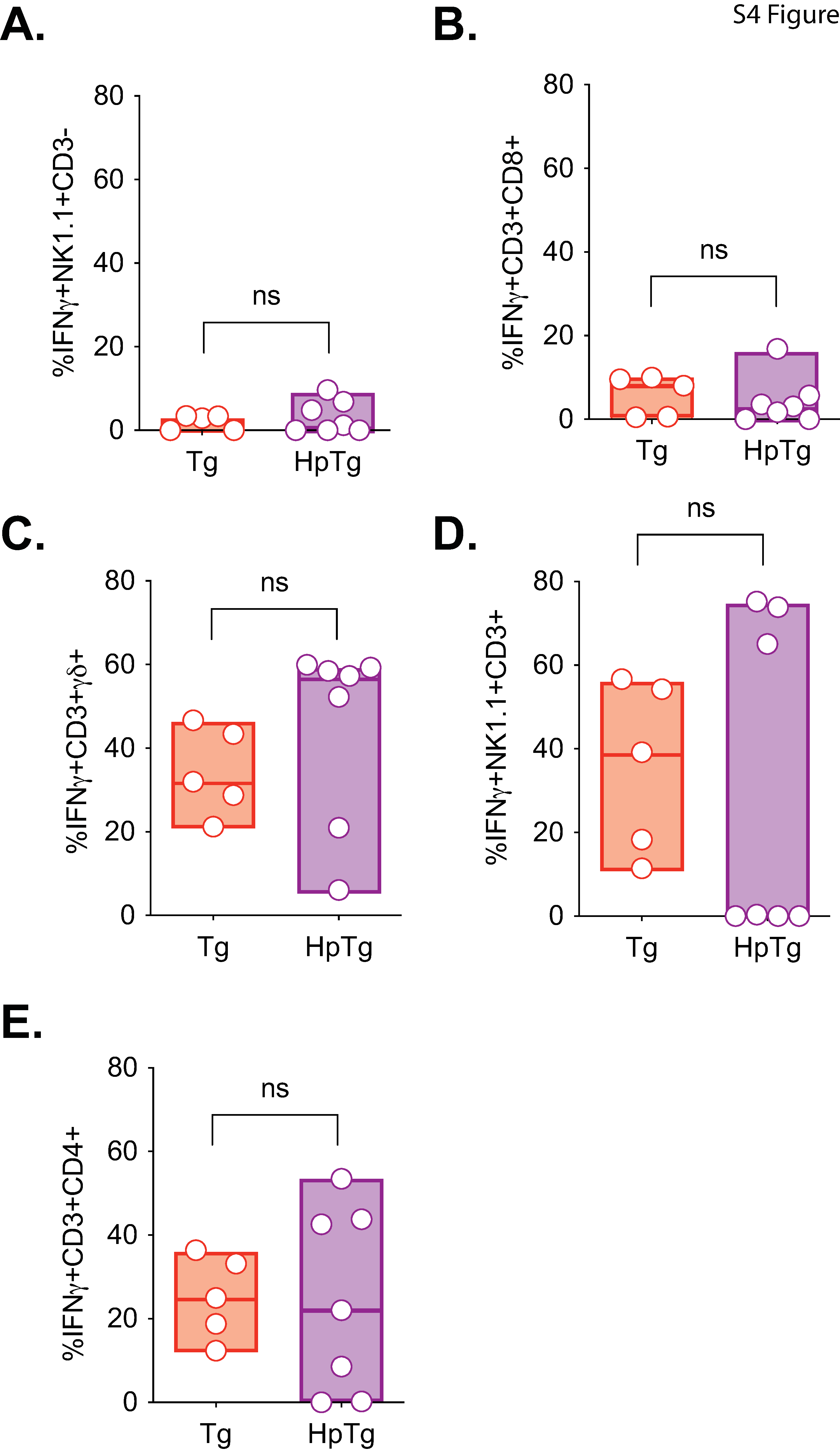

Supplement: S4 Fig — 200 Hb larvae were given orally to mice 7 days prior to infection with 20 Tg tissue cysts. The cell percentage of IFN©-producing (A) NK (IFN©+NK1.1+CD3-), (B) CD8+ T (IFN©+CD3+CD8+), (C) ©δ T (IFN©+CD3+©δ+), (D) NKT (IFN©+NK1.1+CD3+) and (E) CD4+ T cells (IFN©+CD3+CD4+) in Tg and HbTg animals 10 days post Tg infection. N = 2–4 mice per group per experiment, 2 independent experiments. Data was tested for normality. ANOVA or Kruskal-Wallis tests were performed on parametric/non-parametric pooled data including N/Tg/HbTg/Hb groups, and when significant, Sidak’s/Dunn’s Multiple comparisons were performed on Hb vs. HbTg and Tg vs. HbTg; n.s. = non significant. (TIF) [file pone.0292408.s004.tif]

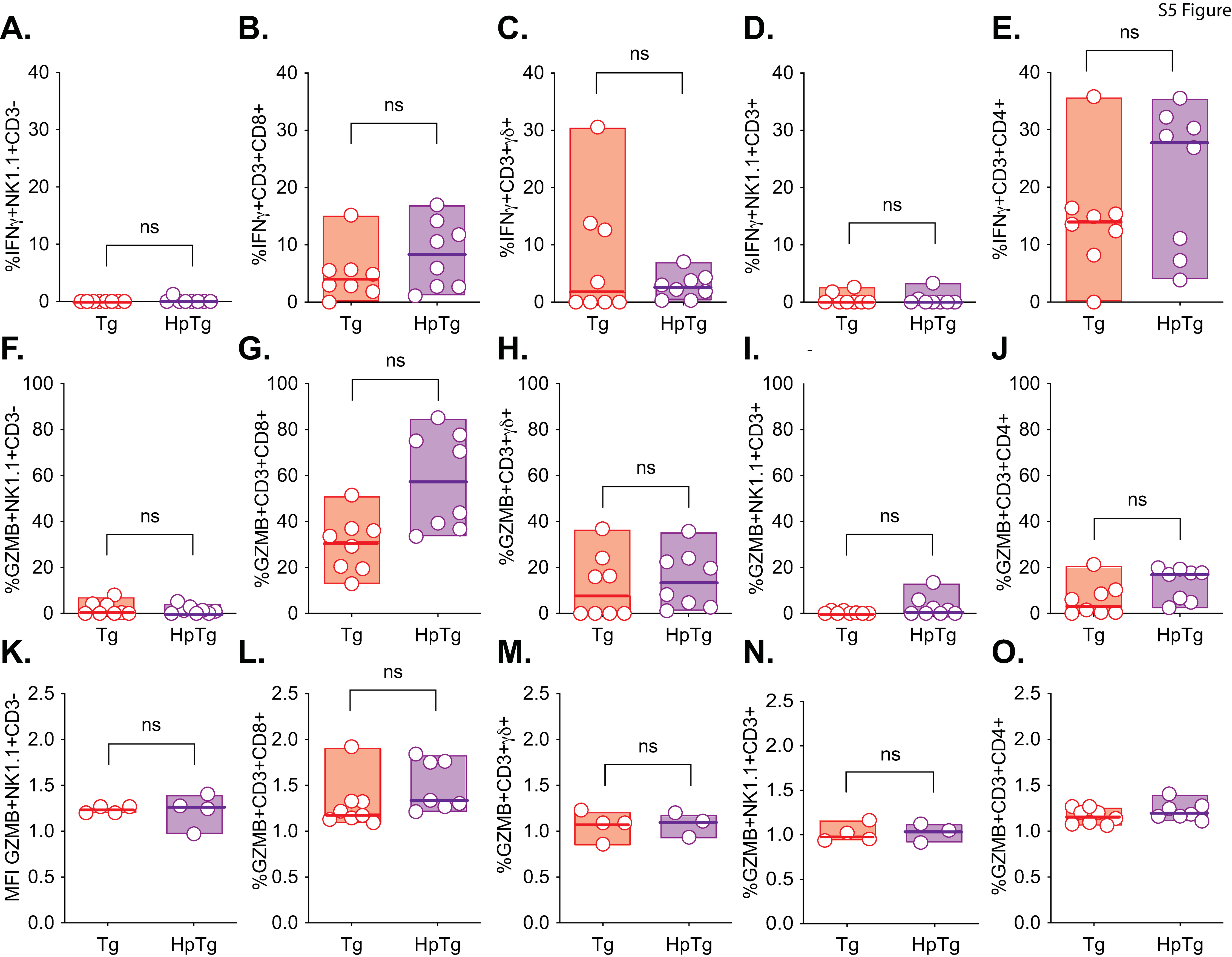

Supplement: S5 Fig — 200 Hb larvae were given orally to mice 7 days prior to infection with 20 Tg tissue cysts. The percentage of IFN©-producing (A) NK (IFN©+NK1.1+CD3-), (B) CD8+ T (IFN©+CD3+CD8+), (C) ©δ T (IFN©+CD3+©δ+), (D) NKT (IFN©+NK1.1+CD3+) and (E) CD4+ T cells (IFN©+CD3+CD4+) in Tg and HbTg animals. The cell percentage of GZMB-producing (F) NK (GZMB+NK1.1+CD3-), (G) CD8+ T (GZMB+CD3+CD8+), (H) ©δ T (GZMB+CD3+©δ+), (I) NKT (GZMB+NK1.1+CD3+) and (J) CD4+ T cells (GZMB+CD3+CD4+) in Tg and HbTg animals. GZMB MFI levels on (K) NK (NK1.1+CD3-), (L) CD8+ T (CD3+CD8+), (M) ©δ T (CD3+©δ+), (N) NKT (NK1.1+CD3+) and (O) CD4+ T (CD3+CD4+) cells relative to naïve animals. N = 2–4 mice per group per experiment, 2 independent experiments. Data were tested for normality. ANOVA or Kruskal-Wallis tests were performed on parametric/non-parametric pooled data including N/Tg/HbTg/Hb groups, and when significant, Sidak’s/Dunn’s Multiple comparisons were performed on Naive vs. Tg and Tg vs. HbTg; n.s. = non significant. (TIF) [file pone.0292408.s005.tif]

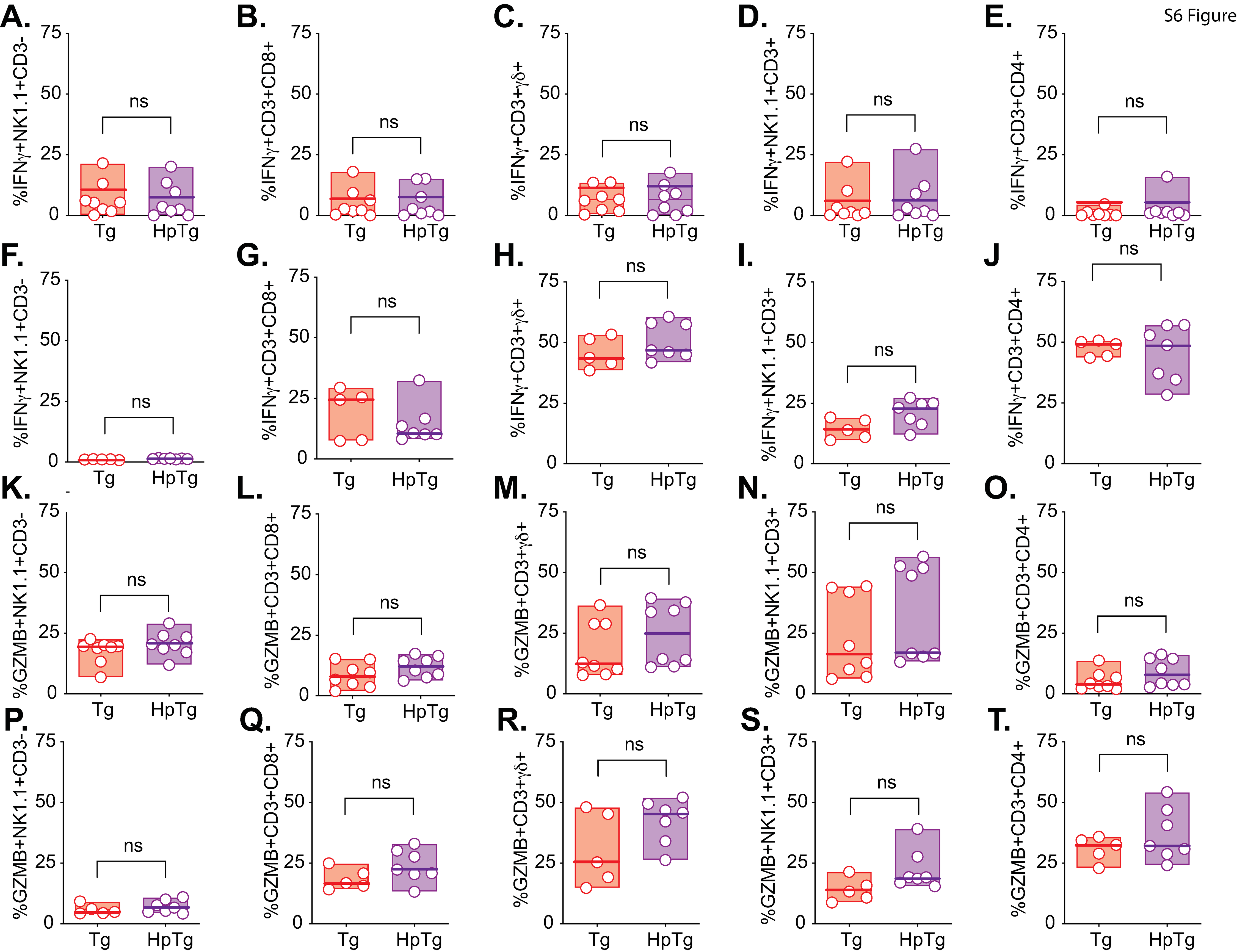

Supplement: S6 Fig — 200 Hb larvae were given orally to mice 7 days prior to infection with 20 Tg tissue cysts. The cell percentage of IFN©-producing (A) NK (IFN©+NK1.1+CD3-), (B) CD8+ T (IFN©+CD3+CD8+), (C) ©δ T (IFN©+CD3+©δ+). (D) NKT (IFN©+NK1.1+CD3+) and (E) CD4+ T cells (IFN©+CD3+CD4+) in Tg and HbTg animals 5 days post Tg infection. The percentage of IFN©-producing (F) NK (IFN©+NK1.1+CD3-), (G) CD8+ T (IFN©+CD3+CD8+), (H) ©δ T (IFN©+CD3+©δ+), (I) NKT (IFN©+NK1.1+CD3+) and (J) CD4+ T cells (IFN©+CD3+CD4+) in Tg and HbTg animals 10 days post Tg infection. The cell percentage of GZMB-producing (K) NK (GZMB+NK1.1+CD3-), (L) CD8+ T (GZMB+CD3+CD8+), (M) ©δ T (GZMB+CD3+©δ+), (N) NKT (GZMB+NK1.1+CD3+) and (O) CD4+ T cells (GZMB+CD3+CD4+) in Tg and HbTg animals 5 days post Tg infection. The cell percentage of GZMB-producing (P) NK (GZMB+NK1.1+CD3-), (Q) CD8+ T (GZMB+CD3+CD8+), (R) ©δ T (GZMB+CD3+©δ+), (S) NKT (GZMB+NK1.1+CD3+) and (T) CD4+ T cells (GZMB+CD3+CD4+) in Tg and HbTg animals 10 days post Tg infection. N = 2–4 mice per group per experiment, 2 independent experiments. Data were tested for normality. ANOVA or Kruskal-Wallis tests were performed on parametric/non-parametric pooled data including N/Tg/HbTg/Hb groups, and when significant, Sidak’s/Dunn’s Multiple comparisons were performed on Naive vs. Tg and Tg vs. HbTg; n.s. = non significant. (TIF) [file pone.0292408.s006.tif]

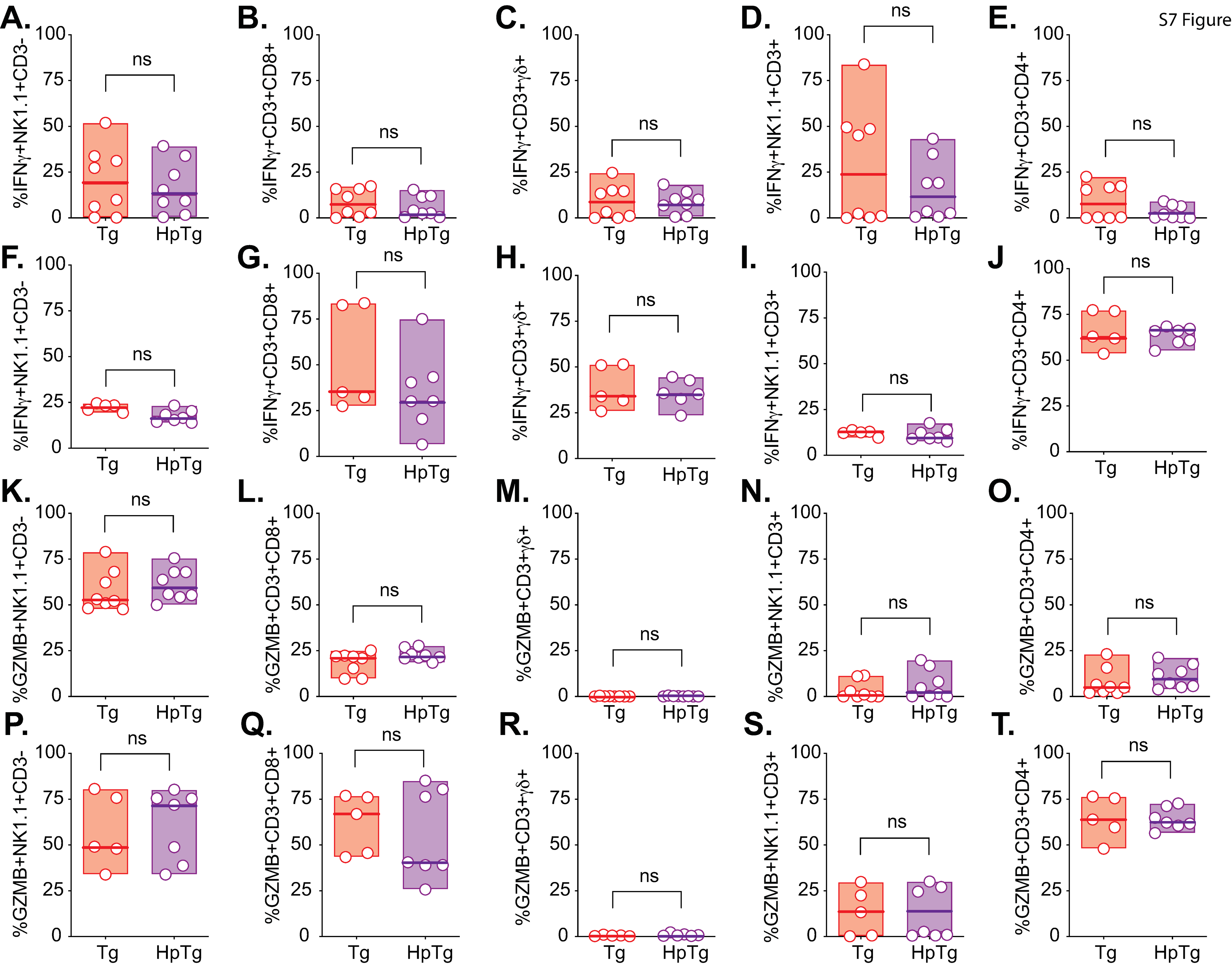

Supplement: S7 Fig — 200 Hb larvae were given orally to mice 7 days prior to infection with 20 Tg tissue cysts. The cell percentage of IFN©-producing (A) NK (IFN©+NK1.1+CD3-), (B) CD8+ T (IFN©+CD3+CD8+), (C) ©δ T (IFN©+CD3+©δ+), (D) NKT (IFN©+NK1.1+CD3+) and (E) CD4+ T cells (IFN©+CD3+CD4+) in Tg and HbTg animals 5 days post Tg infection. The percentage of IFN©-producing (F) NK (IFN©+NK1.1+CD3-), (G) CD8+ T (IFN©+CD3+CD8+), (H) ©δ T (IFN©+CD3+©δ+), (I) NKT (IFN©+NK1.1+CD3+) and (J) CD4+ T cells (IFN©+CD3+CD4+) in Tg and HbTg animals 10 days post Tg infection. The cell percentage of GZMB-producing (K) NK (GZMB+NK1.1+CD3-), (L) CD8+ T (GZMB+CD3+CD8+), (M) ©δ T (GZMB+CD3+©δ+), (N) NKT (GZMB+NK1.1+CD3+) and (O) CD4+ T cells (GZMB+CD3+CD4+) in Tg and HbTg animals 5 days post Tg infection. The cell percentage of GZMB-producing (P) NK (GZMB+NK1.1+CD3-), (Q) CD8+ T (GZMB+CD3+CD8+), (R) NKT (GZMB+NK1.1+CD3+), (S) ©δ T (GZMB+CD3+©δ+) and (T) CD4+ T cells (GZMB+CD3+CD4+) in Tg and HbTg animals 10 days post Tg infection. N = 2–4 mice per group per experiment, 2 independent experiments. Data were tested for normality. ANOVA or Kruskal-Wallis tests were performed on parametric/non-parametric pooled data including N/Tg/HbTg/Hb groups, and when significant, Sidak’s/Dunn’s Multiple comparisons were performed on Naive vs. Tg and Tg vs. HbTg; n.s. = non significant. (TIF) [file pone.0292408.s007.tif]

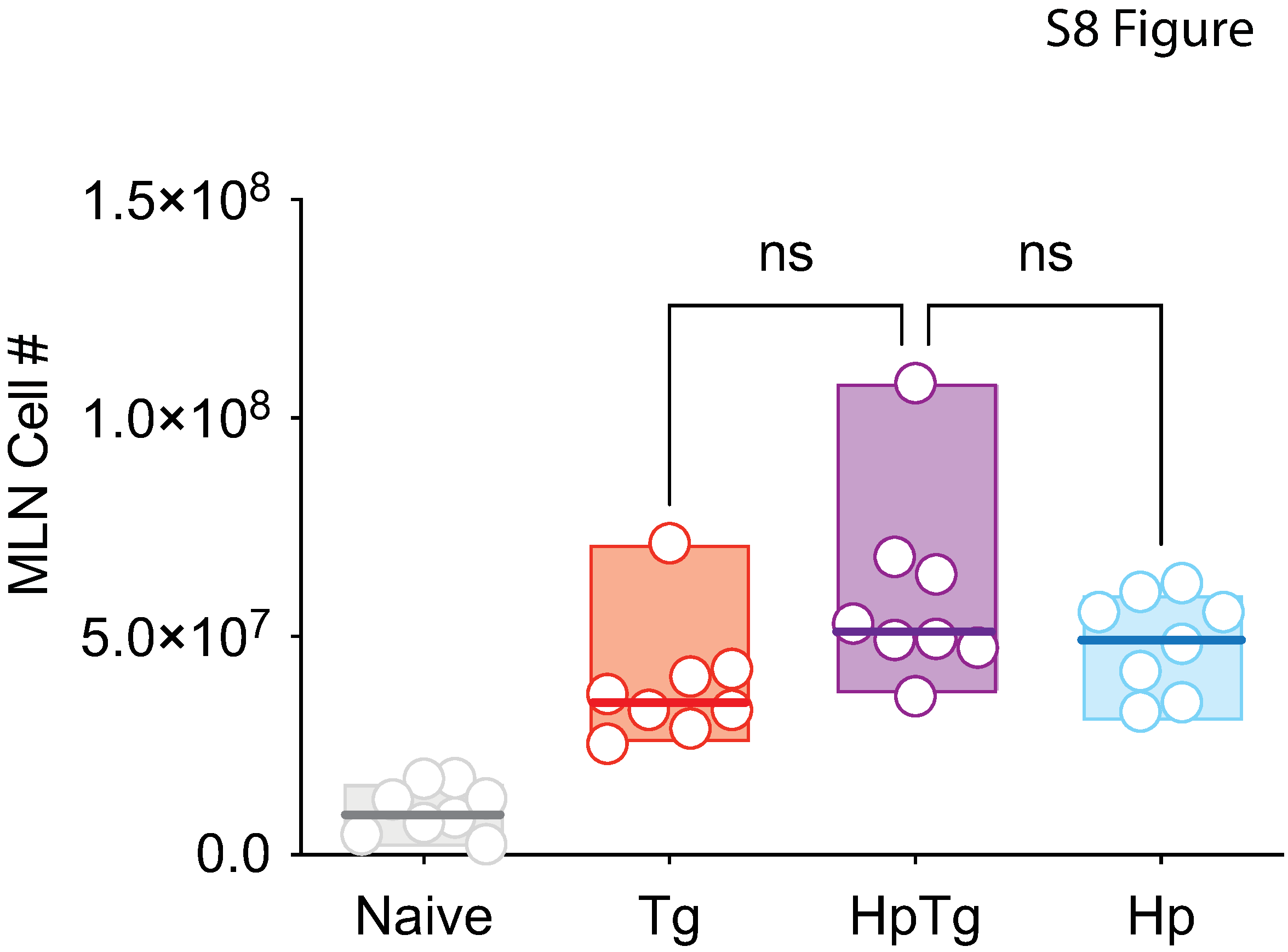

Supplement: S8 Fig — 200 Hb larvae were given orally to mice 7 days prior to infection with 20 Tg tissue cysts. Single cell suspensions were isolated from the mesenteric lymph nodes 5 days post Tg infection and viable cells were counted. Kruskal-Wallis tests were performed on non-parametric pooled data including N/Tg/HbTg/Hb groups, and when significant, Dunn’s Multiple comparisons were performed on Naive vs. Tg and Tg vs. HbTg; n.s. = non significant. (TIF) [file pone.0292408.s008.tif]

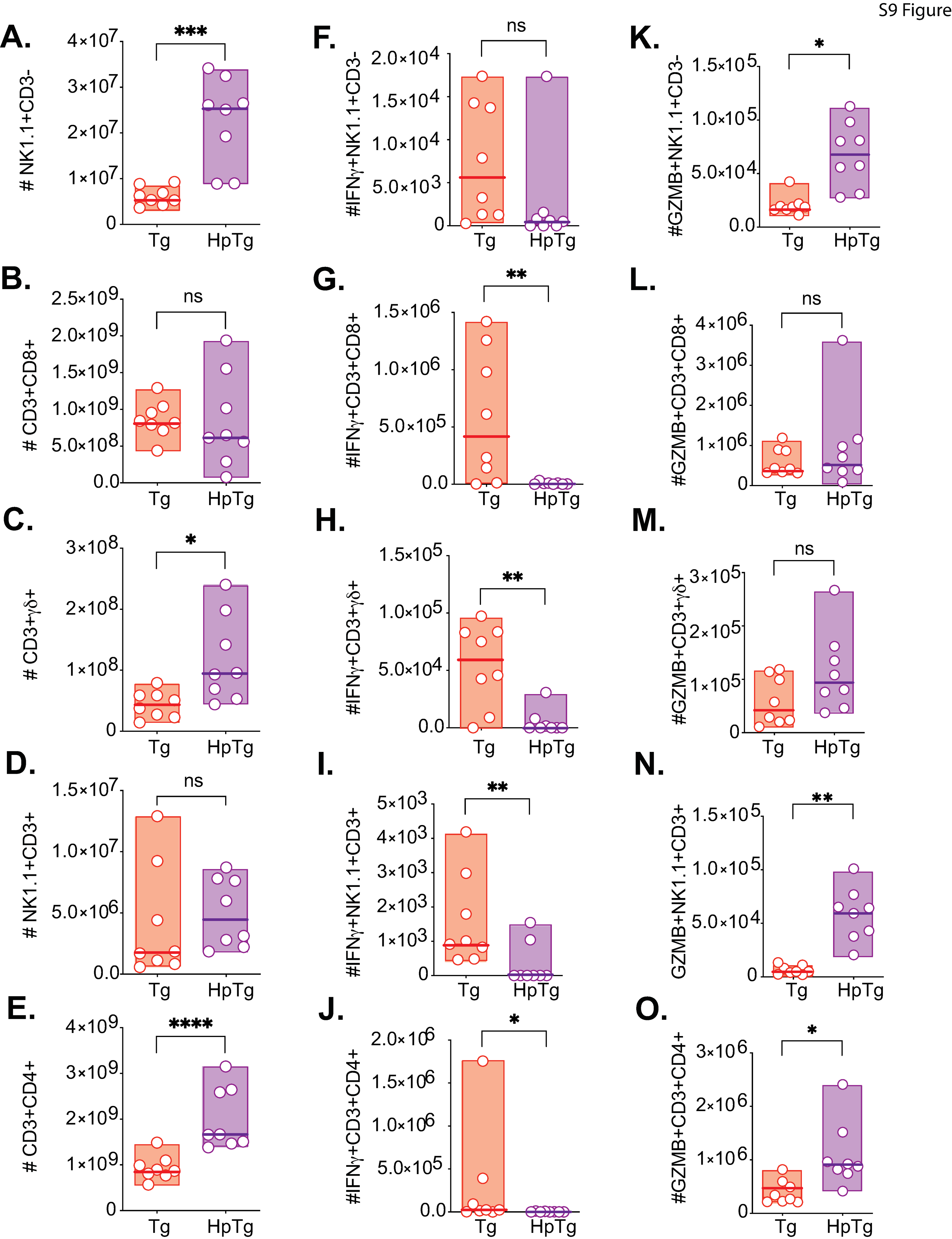

Supplement: S9 Fig — 200 Hb larvae were given orally to mice 7 days prior to infection with 20 Tg tissue cysts. (A-E) The cell number of (A) NK (IFN©+NK1.1+CD3-), (B) CD8+ T (IFN©+CD3+CD8+), (C) ©δ T (IFN©+CD3+©δ+), (D) NKT (IFN©+NK1.1+CD3+) and (E) CD4+ T cells (IFN©+CD3+CD4+) in Tg and HbTg animals. (F-J) The cell number of IFN©-producing (F) NK (IFN©+NK1.1+CD3-), (G) CD8+ T (IFN©+CD3+CD8+), (H) ©δ T (IFN©+CD3+©δ+), (I) NKT (IFN©+NK1.1+CD3+) and (J) CD4+ T cells (IFN©+CD3+CD4+) in Tg and HbTg animals. (K-O) The cell number of GZMB-producing (K) NK (GZMB+NK1.1+CD3-), (L) CD8+ T (GZMB+CD3+CD8+), (M) ©δ T (GZMB+CD3+©δ+), (N) NKT (GZMB+NK1.1+CD3+) and (O) CD4+ T cells (GZMB+CD3+CD4+) in Tg and HbTg animals. N = 2–4 mice per group per experiment, 2 independent experiments. Data was tested for normality (Anderson-Darling test). ANOVA or Kruskal-Wallis tests were performed on parametric/non-parametric pooled data including N/Tg/HbTg/Hb groups, and when significant, Sidak’s/Dunn’s Multiple comparisons were performed on Hb vs. HbTg and Tg vs. HbTg; n.s. = non significant, * = p<0.05 and ** = p<0.01. (TIF) [file pone.0292408.s009.tif]

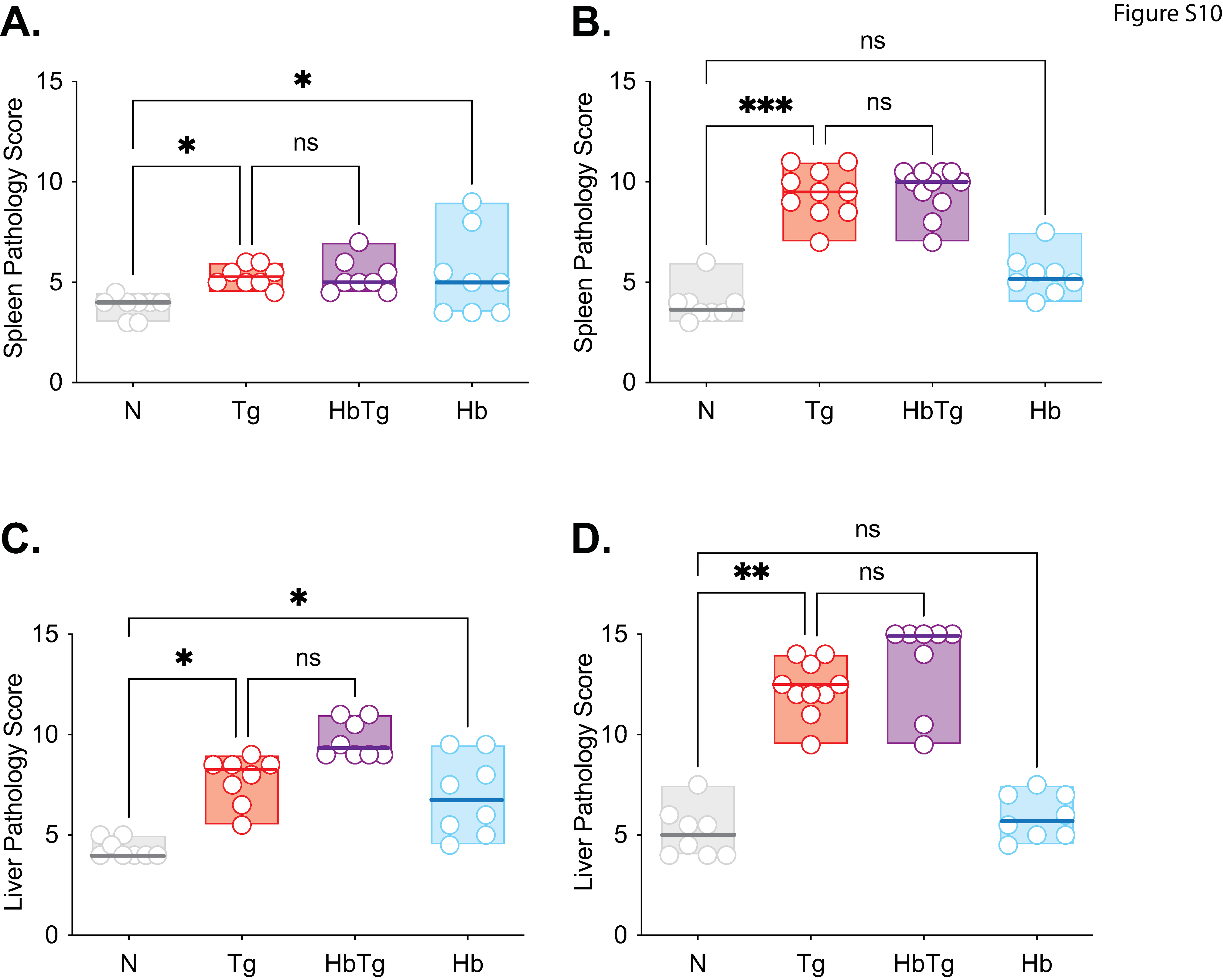

Supplement: S10 Fig — 200 Hb larvae were given orally to mice 7 days prior to infection with 20 Tg tissue cysts. Spleens and livers were harvested from mice 5 and 10 days post Tg infection, and formalin fixed. 6 μM slides were cut from the paraffin embedded swiss rolls, and stained with hematoxylin and eosin. Average spleen (A & B) and liver (C & D) pathology score for each mouse, euthanized at 5 (A &C) 10 (B & D) days post Tg infection. N>3 mice per group per experiment, 2 independent experiments. Data was tested for normality (Anderson-Darling test). ANOVA or Kruskal-Wallis tests were performed on parametric/non-parametric pooled data including N/Tg/HbTg/Hb groups, and when significant, Sidak’s/Dunn’s Multiple comparisons were performed on N vs Tg and Tg vs. HbTg; n.s. = non significant, * = p<0.05 and ** = p<0.01. (TIF) [file pone.0292408.s010.tif]

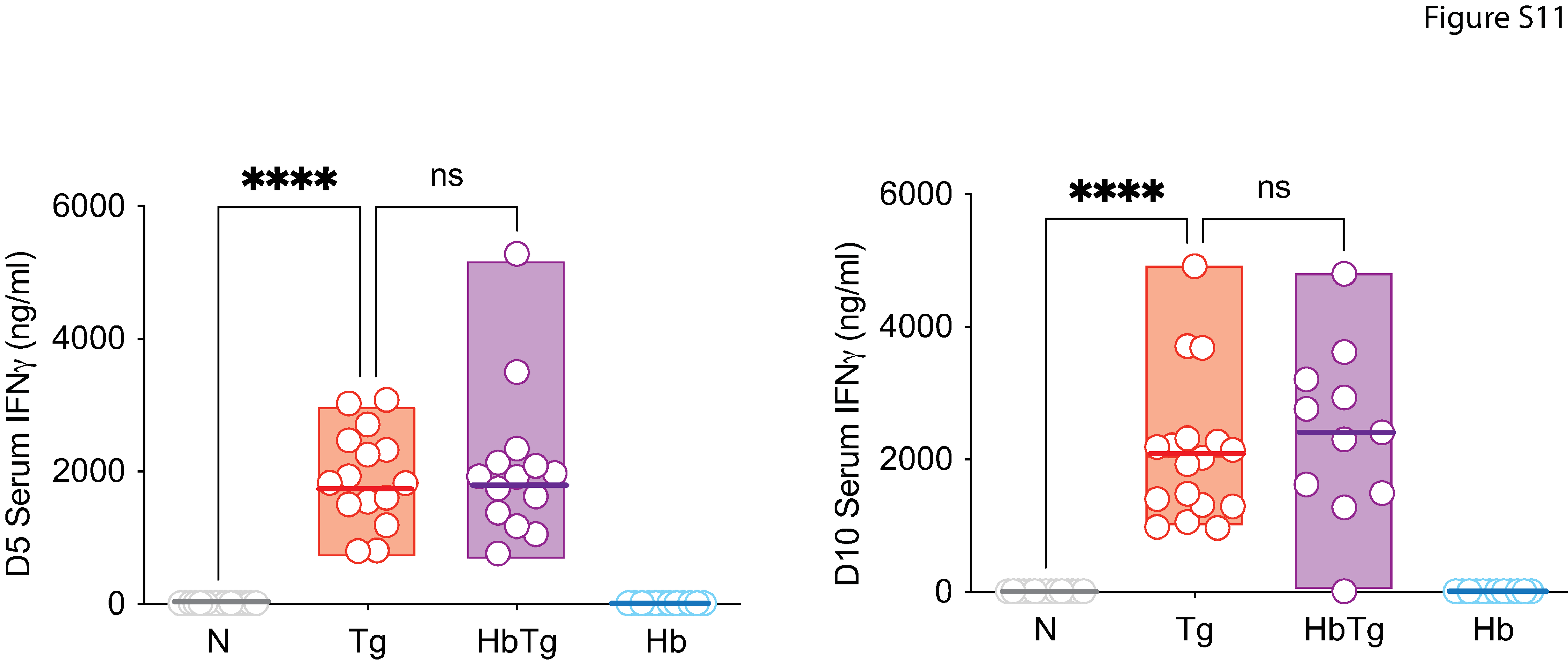

Supplement: S11 Fig — IFN© protein levels were measured in the serum by ELISA at 5 and 10 days post Tg infection. N = 2–4 mice per group per experiment, a minimum of 2 independent experiments. Data was tested for normality (Anderson-Darling test). ANOVA or Kruskal-Wallis tests were performed on parametric/non-parametric pooled data, and when significant, Sidak’s/Dunn’s Multiple comparisons were performed on Hb vs. HbTg and Tg vs. HbTg; **** = p<0.0001. (TIF) [file pone.0292408.s011.tif]
