# Supplementary material for: Heligmosomoides bakeri and Toxoplasma gondii co-infection leads to increased mortality associated with changes in immune resistance in the lymphoid compartment and disease pathology
Source: PLoS One. 2024 Jul 1;19(7):e0292408. doi: 10.1371/journal.pone.0292408 (PMC11216590; doi:10.1371/journal.pone.0292408)
Supplement: Attachment — Submitted filename: Response_to_Reviewers.docx [file pone.0292408.s014.docx]

We wish to thank the reviewers and editor for taking the time to read and review our manuscript. Time for these efforts is difficult to find, and we value your input. We appreciate all the insightful comments which have helped create a better version of our original submission. Please see our detailed response below.

**Journal Requirements:**

1. Please ensure that your manuscript meets PLOS ONE's style requirements, including those for file naming.

*We have changed all file names and updated the bibliography and affiliations to fit with Plos One’s requirements.*

2. We note that the grant information you provided in the ‘Funding Information’ and ‘Financial Disclosure’ sections do not match.

When you resubmit, please ensure that you provide the correct grant numbers for the awards you received for your study in the ‘Funding Information’ section.

*We have provided grant numbers when they exist. For those that do not have external grant numbers (from the funding agency), we have not provided a number.*

3. Thank you for stating the following in the Acknowledgments Section of your manuscript:

"..This works was funded through Dr. Finney’s grants from the Canadian Foundation for Innovation and the Natural Sciences and Engineering Research Council of Canada (NSERC), as well as scholarships for Dr Anupama Ariyaratne (NSERC Create in Host Parasite Interactions), Namratha Badawadagi (University of Calgary Markin scholarship), Kayla Bailey and Emma Forrester (University of Calgary PURE Scholarship), Dr Joel Bowron and Beverly Dong (NSERC), Camila Gaio and Manfred Ritz (Mitacs Globalinks Scholarships), Shashini Perera (Alberta Graduate Excellence Scholarship) and Dr Edina K Szabo (UCalgary Eyes High Postdoctoral Scholarship)."

Funding information should not appear in the Acknowledgments section or other areas of your manuscript. We will only publish funding information present in the Funding Statement section of the online submission form.

Please remove any funding-related text from the manuscript and let us know how you would like to update your Funding Statement.

*We have removed this section.*

Currently, your Funding Statement reads as follows:

"This works was funded through Dr. Finney’s grants from the Canadian Foundation for Innovation and the Natural Sciences and Engineering Research Council of Canada (NSERC), as well as scholarships for Dr Anupama Ariyaratne (NSERC Create in Host Parasite Interactions), Namratha Badawadagi (University of Calgary Markin scholarship), Kayla Bailey and Emma Forrester (University of Calgary PURE Scholarship), Dr Joel Bowron and Beverly Dong (NSERC), Camila Gaio and Manfred Ritz (Mitacs Globalinks Scholarships), Shashini Perera (Alberta Graduate Excellence Scholarship) and Dr Edina K Szabo (UCalgary Eyes High Postdoctoral Scholarship).

The funders had no role in study design, data collection and analysis, decision to publish, or preparation of the manuscript."

Please include your amended statements within your cover letter; we will change the online submission form on your behalf.

*This has been included in the cover letter.*

4. We note that you have included the phrase “data not shown” in your manuscript. Unfortunately, this does not meet our data sharing requirements. PLOS does not permit references to inaccessible data. We require that authors provide all relevant data within the paper, Supporting Information files, or in an acceptable, public repository. Please add a citation to support this phrase or upload the data that corresponds with these findings to a stable repository (such as Figshare or Dryad) and provide and URLs, DOIs, or accession numbers that may be used to access these data. Or, if the data are not a core part of the research being presented in your study, we ask that you remove the phrase that refers to these data.

*All instances of ‘data not shown’ have been replaced with Supporting Figures.*

5. Please upload a new copy of Supporting Figure S5 as the detail is not clear.

*Figure has been amended and uploaded.*

**Reviewers' comments:**

Major comments:
Line 261-262: Here, the authors have mentioned that increased mortality seen in co-infected group was due to the intestinal worm burden only. But, in fig1, except MLN Tg burden in HbTg group at day 10 showed significant differences, but none of the other parameters like granuloma numbers and size and macrophage counts were not showing any differences between the compared groups. It is not clear why the authors think the mortality of animals were due to the Tg burden only, but not other factors? For instance, the mortality could be due to contribution of Tg mediated overt Th1 immune responses and also infiltration of other inflammatory cells at day 10 post infection? Please clarify.

*Apologies for the confusion. Increased mortality at this point in the paper (Fig 1) is being studied in the context of parasite burden (Tg and Hp). We have removed the graphs relating to granuloma cell composition and added them later to the intestinal pathology figures (6 & 7) for clarity. In this later section, we now highlight cell infiltration on day 10 post Tg infection as being associated with HbTg infected animals (lines 545-546).*

Line 297-298: In Fig 1C, it looks like Hb burden from two mice in co-infection group are outliers and that could result significance differences between these two comparisons with p<0.05. Did authors looked the same results even after removing these outliers? if not, please rephrase the sentence here.

*For Fig 1C, data in all 4 groups are normally distributed according to the D’Agostino Pearson normality test.*

*Hb burden is variable. Please see the following as way of example: DOI:*[*10.3791/52412*](http://dx.doi.org/10.3791/52412)*, DOI:* [*10.1084/jem.20101074*](https://www.ncbi.nlm.nih.gov/pmc/articles/PMC2964568/)*,  DOI:* [*10.3389/fimmu.2022.1020056*](https://doi.org/10.3389/fimmu.2022.1020056)

*If we remove both data points and rerun the analysis, the data remain significant.*

Also, the error bars at day10 in Fig 1F seems to be no differences between the groups. Please double check the significance here.

*We have double checked significance. Our values are correct. To better visualise the difference, we have amended the colours on the graph.*

Line 310-314: It seems that figure numbers are mislabeled here. Double check all the figure numbers.

*We have amended the incorrect figure numbers.*

Line 367: Please separate the interpretations for Il-4 and Il-13 expression in the text, because Il-4 expression was not increased even in day 5 in Hb infected animals, but only at day10, both Il-4 and Il-13 expression were increased. Also, rephrase the sentence here by mentioning the differences in the day 5 and day 10 for each cytokine.

*We have now separated the interpretations and mentioned all differences between IL-4 and IL-13 at the 2 different time points (lines 359-372).*

Line 369-370: In the example here, only Il-13 expression was increased, but not Il-4 at day 5pi.

*We have now separated the interpretations and mentioned all differences between IL-4 and IL-13 at the 2 different time points (lines 359-372).*

Line 387 (Fig 3A & 3F): In Fig 3A, it looks like CD4 producing Ifng levels were almost null in all 4 groups, but in Fig 3F, it was shown as CD4 expressing Ifng from Tg group is significantly different than co-infected group. Are these subtle differences coming from the isolated MLN or from whole intestine?

*The data in figure 3 are all from the MLN. This is mentioned in the figure legend and title. We have no flow cytometry data from the intestine since obtaining live lymphocytes for PMA/ionomycin stimulation from parasite-infected intestines proved unreliable. However, the data from the PP (closely linked to the SI) show no difference in IFNg producing CD4 cells, as stated in the text.*

*The Y axis in Fig 3F has also been changed to better visualise the difference between the HbTg and Tg groups.*

Line 471-473: It seems to be that figure numbers are mislabeled here. There is no 5J, 5K-O in the attached main figures.

*Apologies. This was supposed to be labelled SI Fig 5. It has been relabelled appropriately.*

Line 491-493: Here authors have mentioned that “differences in mortality (Fig 1B)” we saw no differences….. But in Fig 1B, the actual differences in mortality between Tg and HbTg starts at day 8, but not at day 5. Hence, we don’t expect any differences between the groups at day 5, but only at day 10. Therefore, day 10 data should be ideal to include as representative figure in the place of day 5, although there were no differences noted at day 10 (as mentioned by authors in the text “data not shown”).

*Although differences in mortality only start at day 8, we would expect immunological/pathology changes before that point that would lead to these differences. For example, in* <https://doi.org/10.4049/jimmunol.2200504> *, a very similar study, they see mortality from day 12 post infection. However, they study immunity prior to mortality (day 3-10). Specifically, they look at IFNg producing cells at day 10, ~2 days before the onset of mortality. This equates to our day 5 time point. Other publications show that Tg infection alone induces an increase in IL-12 and IFNg from day 3 onwards (*[PMID: 7915739](https://pubmed.ncbi.nlm.nih.gov/7915739/) and doi: [10.1371/journal.ppat.1009970](https://doi.org/10.1371%2Fjournal.ppat.1009970)). *We were therefore interested in the day 5 time point.*

*We have added day 10 data (Fig 7) and changed the wording in this section to make the importance of both time points clearer (see section: HbTg infected animals have intestinal pathology characteristic of both Tg and Hb parasites).*

Line 497-506 (In Fig 6): There is no differences seen between Tg and HbTg in the majority of the parameters tested here, except Fig 6H, 6I and 6L. Therefore, the title for this figure description is slightly overstated. If not the inflammation and also Tg burden (partially), then who is contributing the mortality of the animal? Did authors look into the other non-lymphoid compartments in parallel, especially the role of inflammatory monocytes and neutrophils for the death of the animal with necrotic tissues? Although we understand that the aims of this manuscript is to investigate the cytokine expression in the lymphoid compartment, but the parallel lack of interrogation on the contribution of non-lymphoid immune cells is evident. This has become important as the manuscript titled “co-infection leads to increased mortality with intestinal pathology”. Therefore, it is suggested to mention and discuss the potential effects of overt pathology by other immune cells in addition to the lymphoid compartment that could possibly led to mortality (in the limitation of the study section).

*Unfortunately, we did not study non lymphoid cells. However, others have (*[*https://doi.org/10.4049/jimmunol.2200504*](https://doi.org/10.4049/jimmunol.2200504) *) and shown that in a similar infection model to ours (200 Hb 9 days prior to 10 cysts Me49 Tg), early infiltration of inflammatory monocytes and neutrophils to the peritoneal cavity was impaired in HbTg mice and that this may ultimately account for the reduced IFNg production by lymphoid cells. This dysregulation was later restored and did not solely account for differences in mortality.*

*We have now added text to reflect this and changed the title of the section to clarify our findings (lines 608-610).*

Reviewer #2: 

1. Can the authors explain why they chose 7 days post Hb infection to do the Tg co-infection? 7 days is the worst timing for Hb infection. It's not a chronic infection yet, the immune response in the intestine is not established. Why not wait a couple of weeks with Hb infection to then infect with Tg? Or why use a C57Bl/6 mice that is not going to give a strong Th2 response and also will have the worst pathology?

*From previous publications we know that co-infection outcome can vary depending on infection time, length and the order of which parasitic infection established itself first (DOI: 10.3389/fcimb.2017.00341). We specifically wanted to focus on the acute infection phases of both parasites for the following reasons:*

- *Many studies on HbTg co-infection infect animals with Tg as Hb adults emerge and start to reproduce, between 7 and 12 days post Hb infection:* [*doi.org/10.4049/jimmunol.2200504*](https://doi.org/10.4049/jimmunol.2200504) *, DOI:* [*10.1128/IAI.01236-07*](https://doi.org/10.1128/iai.01236-07)*, doi:* [*10.1128/IAI.73.9.5468-5481.2005*](https://doi.org/10.1128%2FIAI.73.9.5468-5481.2005) *, doi: 10.1016/j.cell.2021.01.051,* [*https://doi.org/10.4049/jimmunol.1601741*](https://doi.org/10.4049/jimmunol.1601741)*. To compare our results to theirs, we stayed within this timeframe.*
- *At day 7 post-infection, Hb specific Th2 responses (IL-4 and IL-13) are already measurable in the MLN, where Treg numbers are also significantly increased (DOI: 10.1002/eji.201948392). Granulomas are also visible in the intestinal mucosa and genes involved in monocyte and eosinophil attraction as well as ECM remodelling are increased from 20 to >4000 fold (DOI 10.3389/fimmu.2022.1020056).*
- *Our model represents the situation when people/animals are infected for the first time. While chronic infection offers interesting insights, it is unlikely that people/animals living in endemic regions are infected chronically just once. They are likely infected multiple times while they remain in the same environment (grazing/trickle model – well described for Trichuris muris here:* <https://doi.org/10.1371/journal.ppat.1007926> and for *H. bakeri here DOI 10.3389/fimmu.2022.1020056 and/or drug cure/reinfection model). The impact of these more realistic scenarios at later time points is something of great interest to the group but beyond the scope of this study.*

*We are using C57BL/6 for the following reasons:*

- *From* [*https://doi.org/10.1016/j.pt.2016.11.007*](https://doi.org/10.1016/j.pt.2016.11.007)*: ‘BALB/c mice are less susceptible to infection by oral inoculation with cysts. Brain cysts, a marker of dissemination and chronicity, are either not formed or present in small numbers in BALB/c mice [47,49,57,58].’*
- *Chronic Hb infection establishes itself so it represents a good model for susceptible individuals.*

2. The content written in lines 63 to 70 does not help the point of the research. Inserting this information at that point of introduction is confusing and does not connect with the following paragraphs. Review the necessity of that information, or consider including it after line 105.

*The information has been moved.*

3. Consider adding a conclusion to the introduction section.

*A conclusion has been added.*

4. Can the authors comment on the necessity of stimulating the cells for Flow Cytometry with PMA and Ionomycin? Since the animals are already infected with two pathogens, why stimulated the cells for many hours with a polyclonal stimuli? Wouldn't a non-stimulated approach or a stimulation with parasite antigen be a better approach to the research goal?

*At the start of the project, we optimised all techniques. We first started with no polyclonal stimulus but could not obtain strong reproducible responses. We reverted to using PMA/Ionomycin.*

5. Line 271 - Instead of 16 days post Tg infection, should be Hb infection.

*The text is correct. For survival, days are measured post Tg infection.*

6. Between lines 304 and 314 all references of the figures are wrong. For example, line 304 reference figures 1E & 1F, but it should be 1F & 1G.

*We have amended the incorrect figure numbers.*

7. Figure 1B - Shouldn't it be Days post Hb infection? Also, the reader can't see the survival line for the naive mice.

*The text is correct. For survival, days are measured post Tg infection.*

*The colours have been amended to see both naïve and Hb 100% survival.*

8. In Figure 1 when the authors add the timepoints of D5 and D10 in the x axis, specially bellow Hb data, they are confusing the reader. Considering Hb infection the timepoints should be D12 and D17.

*We have amended the numbers for the graphs relating to Hb parameters.*

9. Figure 1F - There is a typo in the group names. Hp instead of Hb.

*We have corrected the typo.*

10. The authors should evaluate the need of using dots and bars. In some figures like 1F, the presence of the bars can mislead the reader to think that there is no difference between the groups or that there is an increase in the HpTg group.

*The colours and bars have been amended to make it easier to interpret the figures.*

11. Figure 2: Considering the Th2 cytokines, showing the comparison between Hb and HbTg be more useful for the research, since the Hb infection is the one inducing this cytokines?

*We have now added the Hb vs HbTg statistics to the heatmap on Figure 2. We have kept the bar graphs as they are since we want to highlight comparisons between the Tg and HpTg groups that could be implicated in their survival differences.*

12. Could the lower levels of IFN in the HbTg group (D5) be related to the actual increase in the levels of Th2 cytokines for the same timepoint? Can the author comment about that?

*This is a supposition which hasn’t been directly proven. To date, in* [*https://doi.org/10.4049/jimmunol.2200504*](https://doi.org/10.4049/jimmunol.2200504)*, the authors state that:*

- *‘Taken together, these results suggest that coinfection with* H. polygyrus *impairs the* T. gondii*–induced innate chemokine and cellular responses, resulting in a blunted production of early IFN-γ’.*
- *And ‘It should be noted that previous studies have shown differential activation of neutrophils during helminth infection, with increased expression of IL-13 and other type 2 markers (59). Future studies should examine whether this altered activation state may also be compromising the neutrophil’s capacity to be effectively recruited into the site of* T. gondii *infection’.*

*We have now added text on this in the discussion (lines 603-604).*

13. Do the authors think it's relevant for the paper to show the levels of just IFN in the blood? It makes the reader curious about the systemic level of all cytokines, and we don't see this result.

*The data have been removed from the results section and added as a supplementary figure (S11 Fig). The data are only referred to in the discussion when directly discussing IFNg serum production.*

14. Back to PMA stimulation, do the authors think that the levels of IFN/GranzymeB measured by flow could be misrepresented since the cells could be exhausted?

*This is an interesting suggestion. Exhaustion is usually associated with T cells and chronic situations (chronic infections, cancer etc) which is why we didn’t consider it here. We have now added text in the discussion referring to this (lines 623-627).*

15. Where are Figures 5J to 5O?

*Apologies. This was supposed to be labelled SI Fig 5. It is now SI Fig 9.*

16. Line 468 - Since there is a difference in the MLN number of cells it would be important to support the data from Figures 3 and 4, to show the difference and also the analysis considering the number of cells and not only the %.

*The data are in now S9 Fig.*

17. Considering the results presented in Figure 6 do the authors think that the tittle of the paper is appropriate? Is the increase in mortality associated with pathology, since there is no difference between HbTg and Hb?

*The title of the paper has now been changed and the section on pathology has been clarified (see section: HbTg infected animals have intestinal pathology characteristic of both Tg and Hb parasites).*

18. General comment about the results: It could be useful to expand/show the analysis considering all the groups and timepoints. For example, we don't see the comparison with the groups and the naive mice in all figures, also the analysis between the co-infection group considering the differences between D5 and D10 could increase the depth of the results.

*Many more data have now been added which include comparisons with naïve groups and D10 data. These are also discussed throughout the text.*
